# Health of Louisville

**Published:** 1851-10

**Authors:** 


### HEALTH OF LOUISVILLE.

Since we wrote our last paragraph on this subject we have uot heard of a case of cholera in the city, nor has a case been reported by the Board of Health. In the time, we have experienced some intensely hot weather, the thermometer in the shade having risen several days as high as 93°. No rain has fallen in six weeks. Dysentery, which has been so fatal in many parts of the country, has rather declined in the city during the last month, and with the exception of intermittent fever, very little sickness prevails.

